# Neuroimaging patterns of anatomical features in pediatric cerebral palsy patients at Ayder hospital, Mekelle, Ethiopia

**DOI:** 10.1371/journal.pone.0241436

**Published:** 2020-11-04

**Authors:** Peter Etim Ekanem, Anne Caroline Kendi Nyaga, Elizabeth Akitsa Imbusi, Regina Ekanem, Berhanu Mebrahte, Adhanom Gebreslasie, Nissi Peter

**Affiliations:** 1 Department of Anatomy, College of Health Sciences, Mekelle University, Mekelle, Ethiopia; 2 Department of Pediatrics and Child Health, College of Health Sciences, Mekelle University, Mekelle, Ethiopia; 3 College of Health Sciences, Mekelle University, Mekelle, Ethiopia; Nathan S Kline Institute, UNITED STATES

## Abstract

**Background:**

Neuroradiological studies have greatly improved the knowledge and diagnoses of cerebral palsy with its underlying pathology, types and accompanying changes in brain morphology. However, there is no published study on cerebral palsy neuroimaging patterns in Ethiopia.

**Methods:**

Retrospective chart and neuroimaging reviews were conducted among pediatric patients, who attended Ayder Comprehensive Specialized Hospital between January 2016 and August 2019, fulfilling the study criteria. The magnetic resonance images and computed tomography scans reviewed by a neuroradiologist and/or pediatric neurologist were included. Data was collected using a structured checklist and analyzed using SPSS statistical software version 22. Results were represented using tables, graphs and images.

**Results:**

The median age at neuroimaging was 2 years. There were more males (54.5%) than females (45.5%) with a male: female ratio of 1.2:1. Majority of the patients had magnetic resonance (81.8%) as opposed to computed tomography scans (18.2%). Most of the patients (69.7%) had been born at term with spastic quadriplegia (33.3%) found to be the leading type of cerebral palsy. 30.3% of the patients had normal neuroimaging studies whereas 69.7% had neuroimaging abnormalities. Anomalies included pathologies of the white matter (18.2%), basal ganglia (15.2%), cortex and lobes (27.3%), corpus callosum (6.1%), lateral ventricles (12.1%), cysts (18.2%) and cerebellum (3%), respectively. Other findings were seen in 45.5% of the patients.

**Conclusion:**

Severe forms of cerebral palsy (spastic quadriplegia) were most common with majorly cortical and subcortical brain involvement.

## Background

Cerebral palsy (CP) is a term used to describe a wide spectrum of non-progressive motor disabilities resulting from brain damage at prenatal and/or perinatal periods of development. It is one of the commonest forms of severe disability occurring in childhood especially after preterm birth [1]. The disability produced by CP can be classified pathophysiologically into four major types: spastic, often resulting from cortical (pyramidal) insults; athetoid /dyskinetic, due to basal ganglia (extrapyramidal) insults; hypotonic, which is more commonly from cerebellar lesions, and mixed [2, 3]. The average prevalence of CP is reported as 2 per 1000 live births. Increased prevalence is observed among very low birth weight and preterm children (100-fold higher) than normal weight and term babies [4, 5]. In addition to motor manifestations, children with CP frequently exhibit cognitive and sensory impairments, epilepsy, and feeding difficulties among others. Except in the mildest cases, CP has a substantial impact on families’ well-being and societal health care costs [6].

Environmental and/or genetic insults impacting on the brain may cause different patterns of structural abnormalities in CP. The outcomes of these events depend mostly on the timing and stage of brain development [7]. These events and timing include^:^ primary neurulation (weeks 3–4 of gestation), prosencephalic development (months 2–3 of gestation), neuronal proliferation (months 3–4 of gestation), neuronal migration (months 3–5 of gestation), organization (month 5 of gestation to years), postnatal development and myelination (birth to years of postnatal development) [8]. The first and second trimesters are therefore, the most critical times for brain development. These are characterized by sequential and sometimes overlapping steps of proliferation, migration and organization as well as myelination. During the 3rd trimester, growth and differentiation events are predominant. Disturbances of brain development during this period, therefore, cause lesions that differ from those caused by earlier insults or developmental disorders [9].

Cerebral palsy is considered to be due to an insult to the developing brain in the fetal, neonatal and/or infancy periods. Other diseases specific to the peripheral nerves of the spinal cord including: spinal muscular atrophy, myelomeningocele or muscular dystrophies, which may cause early motor abnormalities, are not considered under CP [9]. In the era before computed tomography (CT) and magnetic resonance imaging (MRI), anatomical studies of CP were limited and these were primarily associated with unavailability and limited capabilities of neuroimaging. With the development of CT scan, radiographic revelation of brain anatomy and correlation with clinical data especially in regard to prenatal, perinatal, and postnatal brain mal-development became possible. This was particularly with respect to morphologic changes of the cerebral white matter [8].

Neuroradiologic studies have greatly improved the knowledge and the diagnoses of CP with its underlying pathology. These are progressing as imaging techniques are also advancing. The development of modern neuroimaging technology and radiographic correlations with clinical data has also broadened the scope of studies regarding the various types of CP besides accompanying changes in brain morphology. Qualitative neuroimaging studies indicate that spastic CP mainly presents in periventricular leukomalacia (PVL). Dyskinetic CP is associated with damage to the cortical grey matter as well as the basal ganglia and thalamus. CT scan sensitivity, however, is limited in several cases of cerebral palsy. However, it is still very useful especially in limited resource settings where there are no sophisticated imaging techniques to supplement clinical diagnoses of CP. Countries such as Côte d’ Ivoire, Mali, and Sierra Leone among others were reported to lack imaging facilities. They only depended on clinical symptoms for the diagnosis of CP. Recently some of these countries have reported the use of CT as the only available neuroimaging technology but has greatly enhanced the diagnosis of CP [10]. It is important to note, however, that despite its availability and relatively cheaper cost when compared with MRI, CT scan imaging delivers 100–500 times higher ionizing radiation dosages than conventional radiography [11]. Studies have shown that children are at higher risk of radiation-induced carcinogenesis [12]. A study from the UK showed that children who received an ionizing radiation brain dose of ≥50 mGy were 2.8 times more likely to develop brain cancer [13]. It is, therefore, important to weigh the risk versus benefit when determining what imaging modality to use in children even in resource-limited settings.

MRI is more sensitive than CT scan in the detection of gross brain malformations and mild degrees of white matter damage. The important roles of MRI are identification of the anatomical features that could predict adverse neurodevelopmental outcomes and provision of accurate information on the nature of cerebral injury in the population. Therefore, MRI of brain is the neuroimaging study of choice because it defines cortical and white matter structures and abnormalities more clearly than CT scans. The capability of MRI to predict neurodevelopmental outcomes and improve diagnosis of CP has been demonstrated in various studies. Among these is the European Cerebral Palsy study [5] where the children were classified according to the brain injury diagnosed using MRI. This group used a classification system based on the presumed timing and nature of insult that resulted in CP. This included both genetic and non-genetic etiologies such as genetic cortical malformations and hypoxic ischemic injury [5, 6]. Anderson et al. also demonstrated in their study that structured evaluation of brain MRI at term equivalent is predictive of outcome at 7-years of age, independent of clinical and social factors [14]. A review by de Vries et al also stated that MRI is a better tool to predict outcome in the term infant with hypoxic-ischaemic encephalopathy or neonatal stroke. Diffusion-weighted imaging was also included as an additional sequence that adds to the predictive value for motor outcome [15].

Despite the significant contribution of neuroimaging in CP diagnoses reported in developed countries, data reflecting the actual magnitude of CP in low-income countries and Ethiopia in particular, is limited. In one report, doctors from 22 countries in Africa and representatives from a further 5 countries outside Africa participated in discussions around issues affecting the identification and management of children with CP in Africa. Ethiopia was included among the countries that had no ‘*system for at risk babies’*. It was also reported in the same article that only three of the countries mentioned, including Ethiopia, had introduced the use of CT scan machines by 2013 [10].

Ayder Comprehensive Specialized Hospital (ACSH) is one of the few referral hospitals in Ethiopia and the only hospital in Tigray state that offers MRI services. MRI was inaugurated in ACSH in 2015. It is thus a relatively new and expensive procedure. This limits affordability among the general (mainly middle and low-income) population, who pay out of pocket for medical services. The recommendation of the American Neurology Association [9] that every patient with CP should undergo neuroimaging, more so MRI, is not implemented routinely in all patients with CP in ACSH. Rather, the practice leans towards clinical diagnosis of CP with neuroimaging reserved for unclear etiology or clinical course. Even this, however, is limited by affordability. Considering the great economic burden already posed by the long term management and follow up of CP patients on affected families, most families or caregivers are unable to pay for these services. This creates a limitation in the number of CP patients undergoing neuroimaging and consequently availability of data in the region. In view of this, we studied both CT scan and MRI images retrospectively to identify the anatomical patterns of brain morphology in pediatric patients with CP attending ACSH, Mekelle, Tigray region, Ethiopia.

## Materials and methods

This research was conducted in ACSH, a referral hospital found in Mekelle city, Tigray state. It is the second largest hospital in Ethiopia. It was established in 2008 and serves a catchment population of over 8 million people including but not limited to Tigray, Afar and South-eastern part of Amhara states. The study was approved by the Ethics Review Committee of Mekelle University [ERC 1360/2019]. Permission to access patients’ files and images was granted by the Ethics Review Committee and the medical director, Ayder Comprehensive Specialized Hospital, Ethiopia. Retrospective chart and neuroimaging review were employed in this study. Thus, consent was not obtained since the data was analyzed anonymously.

The data for this study was collected between April 2019 and August 2019. Purposive sampling was undertaken to determine the neuroimaging patterns of CP among pediatric patients attending ACSH between January 2016 and August 2019. Included patients were aged less than 18 years with a clinical diagnosis of CP (score of less than or equal to four of five on the Medical Research Council Scale (MRCS) for Muscle Strength in at least one limb with presumed central origin of weakness). They also needed to have CT or MRI scans in the hospital’s computerized radiology system having been reviewed by a neuroradiologist and/or pediatric neurologist. An adapted structured checklist was used to extract data from registration books and medical records. Patients with presence of one or more of: history of malignancy, primary neuromuscular disorder, evidence of developmental regression except in cases of CNS infection in infancy, a known genetic syndrome and brain images not found in the hospital’s radiology system or not reviewed by a neuroradiologist or neurologist were excluded. From the above criteria, a total of 33 patients were included in the study.

MRI studies included sagittal and axial T1-weighted images, 400-800/ 20-35/ 1–2 (TR/ TE/ excitations), and axial T2-weighted images, 2000-3000/ 30-120/ 1–2. CT scans consisted of unenhanced and contrast enhanced CT scans with a single detector scanner (Neusoft, Philips 2007), using 3mm-thick slices. The images were reviewed by two of the authors with knowledge of the preceding clinical diagnosis of CP but without knowledge of the specific clinical manifestations of each case, thus the review was partially blinded. The neuroimaging studies were anatomically evaluated for developmental or acquired abnormalities. These included the corpus callosum, deep and peripheral white matter, cerebral cortex, basal ganglia/thalami, and brainstem anomalies besides malformations such as the presence of parenchymal cysts, hydrocephalus, and ventricular contour defects.

Data was analyzed using IBM SPSS statistical software version 22. Variables studied were: age at imaging, gestation at birth, imaging modality and CT scan or MRI findings.

## Results

As seen in Table 1, abnormalities found in various parts of the brain included:

**Table 1:** Patient characteristics and neuroimaging findings of pediatric patients attending ACSH (January 2016-August 2019).

Case No.	**Sex**	**Age at Imaging**	**Imaging Type**	**Gestation at delivery**	**Clinical Findings**	**Neuroimaging Findings**							
						**White matter**	**Basal Ganglia/ thalamus**	**Cortex/lobes**	**Corpus Callosum**	**Lateral Ventricle**	**Cysts**	**Cerebellum**	**Other findings**
**1**	M	10 m	MRI	Term	Left hemiplegia	Focal deep white matter subcortical infarcts	Normal	Normal	Normal	Normal	No	Normal	-
**2**	F	1yr	MRI	ND	Left hemiparesis	Normal	Normal	Normal	Normal	Normal	No	Normal	-
**3**	M	5 yr	MRI	Term	Hypotonic	Normal	Normal	Normal	Normal	Normal	No	Normal	-
**4**	M	1yr	CT	ND	Spastic quadriplegia	Normal	Normal	Bilateral frontal lobe hypointensity	Normal	Normal	No	Normal	-
**5**	F	4yr	CT	ND	Right Hemiplegia	Normal	Well defined hypoechoic lesion over L. caudate nucleus & anterior limb of internal capsule	Normal	Normal	Normal	No		-
**6**	F	10 m	MRI	ND	Spastic quadriplegia	Normal	Normal	Normal	Normal	Normal	No	Normal	-
**7**	M	5yr	MRI	Term	Left Spastic hemiplegia	Normal	Normal	Normal	Normal	Normal	Arachnoid Cyst (non- pathological)	Normal	-
**8**	M	1yr 8m	CT	ND	Spastic quadriplegia	Normal	Normal	Normal	Normal	Normal		Normal	Minimally increased CSF spaces
**9**	M	3 m	CT	Term	Spastic quadriplegia	Loss of white matter with hypointensities	Normal	Hypodense + patchy enhancement	Normal	dilated	No	Normal	Dilated 3^rd^ ventricle
Case No.	**Sex**	**Age at Imaging**	**Imaging Type**	**Gestation at delivery**	**Clinical Findings**	**Neuroimaging Findings**							
						**White matter**	**Basal Ganglia/ thalamus**	**Cortex/lobes**	**Corpus Callosum**	**Lateral Ventricle**	**Cyst**	**Cerebellum**	**Other findings**
**10**	M	2yrs	MRI	Term	Spastic quadriplegia	Intervening into the gray matter		Bilateral cortical band of grey matter with intervening white matter. -Frontal nodules similar to grey matter	Normal	Normal	No	Normal	Polymicrogyria (bilateral frontal lobes and persylvian fissure)
**11**	M	4yrs	MRI	Term	Choreoathetoid	Normal	Normal	Normal	Normal	Normal	No	Normal	-
**12**	F	1yr 6m	MRI	Term	Spastic quadriplegia	Normal	Normal	Normal	Thinned	Normal	No	Normal	Periventricular hyperintensities
**13**	F	3yrs	MRI	Term	R. Hemiparesis	Normal	Normal	Left frontoparietal and Rt. Frontal atrophy	Normal	Normal	No	Normal	-
**14**	F	2yrs	MRI	Term	Spastic quadriplegia	Normal	Normal	Normal	Normal	Normal	Rt. Temporal porencephalic cyst (pathological)	Normal	Rt parietal old hemorrhage
**15**	F	4yrs	MRI	Term	Hypotonic CP	Normal	Normal	Normal	Normal	Normal	No	Normal	-
**16**	M	4yrs	MRI	ND	Spastic Quadriplegia	Diffuse hyperintensities	Normal	Bilaterally symmetrical subcortical hyperintensities	Normal	Normal	No	Normal	Dilated prevascular spaces on T2 flair
**17**	M	7m	MRI	Term	left hemiparesis	Normal	Normal	Normal	Normal	Normal	No	Normal	Let remarkable DWI restrictions/ADC hypointensities MCA territory
**18**	F	3yrs	MRI	Term	Spastic diplegia	Bilateral deep white matter linear T2/T2 FLAIR hyperintensities with no restriction and no post contrast enhancement	Normal	Normal	Normal	Normal	No	Normal	Bilateral periventricular white matter linear T2/T2 FLAIR hyperintensities with no restriction and no post contrast enhancement
Case No.	**Sex**	**Age at Imaging**	**Imaging Type**	**Gestation at delivery**	**Clinical Findings**	**Neuroimaging Findings**							
						**White matter**	**Basal Ganglia/ thalamus**	**Cortex/lobes**	**Corpus Callosum**	**Lateral Ventricle**	**Cyst**	**Cerebellum**	**Other findings**
**19**	M	3yrs	MRI	Term	Unspecified	Normal	Bilateral T2 FLAIR hyperintensities Bilateral thalamic hyperintensities	Normal	Normal	Normal	Left choroid fissure cyst (non- pathological)	Normal	-
**20**	M	13yrs	MRI	Term	Unspecified	Normal	Normal	Cortical and subcortical atrophy	Normal	Normal	No	Normal	Increased subarachnoid space and adjacent T2 hyperintensity
**21**	F	2yrs	MRI	Term	Hypotonia	Normal	Normal	Normal	Normal	Normal	No	Normal	-
**22**	M	1yr	MRI	Term	Spastic quadriplegia	Normal	Normal	Loss of brain parenchyma mainly bi occipital lobes	Normal	Normal	Cystic changes, which follow CSF, signal intensity sparing only the posterior fossa structures	Normal	Dilatation of 3^rd^ ventricle with mega cisterna magna
**23**	F	10yrs	CT	ND	Left spastic hemiplegia	Normal	Normal	Widening of sulci and prominence of gyri over the Rt. Cerebral hemisphere (frontotemporal and parietal) with volume loss of the right temporal lobe	Normal	Normal	No	Normal	-
**24**	F	15yrs	CT	ND	Unspecified	Normal	Normal	Normal	Normal	Normal	No	Normal	Absent septum pellucidum with squaring of the frontal horns
**25**	M	5m	MRI	ND	Hypotonia	Normal	Normal	Normal	Normal	Normal	No	Normal	-
**26**	M	1.5yr	MRI	Term	Spastic quadriplegia	Normal	Normal	Normal	Normal	Normal	No	Marked paracerebellar atrophy	4^th^ ventricle ex-vacuo dilation and dilated CSF cisterns
**27**	M	1yr 2mo	MRI	Term	Monoparesis	Normal	Normal	Normal	Normal	Normal	No	Normal	-
Case No.	**Sex**	**Age at Imaging**	**Imaging Type**	**Gestation at delivery**	**Clinical Findings**	**Neuroimaging Findings**							
						**White matter**	**Basal Ganglia/ thalamus**	**Cortex/lobes**	**Corpus Callosum**	**Lateral Ventricle**	**Cyst**	**Cerebellum**	**Other findings**
**28**	M	2mo	MRI	Term	Spastic quadriplegia	Normal	Normal	Normal	Normal	Dilated	Multiple cystic lesions with ring enhancing lesions over the bilateral cerebral hemispheres. (pathological)	Normal	Dilated third ventricles. Difficult visualization of brain parnechyma
**29**	F	4yr	MRI	Preterm (7 mo)	Spastic diplegia	Normal	Partial fusion of thalami inferiorly.	incomplete separation of cerebral hemisphere anteriorly	partial hypoplasia	Rudimentary falx & R. occipital horn of the R. ventricle	dorsal interhemispheric cyst (pathological)	Normal	Absent septum pellucidum
**30**	F	3 yr	MRI	Term	choreoathetoid	Normal	Bilateral posterior hyperintensity	Bilateral frontal lobe volume loss compared to the temporal-parietal and occipital formations	Normal	Normal	No	Normal	-
**31**	M	11 m	MRI	Term	Spastic diplegia	Normal	Normal	Normal	Normal	Deep right occipital horn posteriorly. Temporal/frontal minimal increment in CSF spaces including cisterns	No	Normal	-
**32**	F	7yr	MRI	Term	unspecified	Bilateral frontal and occipital lobe T2 FLAIR white matter hyperintensity	Normal	Normal	Normal	Normal	No	Normal	parenchymal volume loss with widening of the interhemispheric fissure and sulci
**33**	F	7yr	MRI	Term	Right Spastic hemiparesis	Normal	Normal	Left MCA region Chronic infarction	Normal	Normal	No	Normal	-

L.: Left R.: Right MCA: middle cerebral artery. CSF: Cerebrospinal fluid m: months yr: years

### White matter abnormalities

Among the 6 patients (18.2%) with white matter anomalies, white matter hyperintensities were observed in 3 patients. Diffuse hyperintensities were seen in case 16, deep white matter linear hyperintensities on T2/T2 FLAIR images in case 18 and bilateral frontal and occipital lobe T2 FLAIR white matter hyperintensities in case 32. Focal deep matter subcortical infarcts were also noted in case 1, white matter loss with diffuse hypointensities in case 9 and white matter intervening into the grey matter was found in case 10.

### Basal ganglia and thalamus abnormalities

4 (12.1%) patients were found to have basal ganglia anomalies. Case 5, who had right-sided spastic hemiplegic CP presented with well-defined hypoechoic lesion over the left caudate nucleus & anterior limb of the internal capsule. Case 19 also had bilateral basal ganglia posterior T2 FLAIR hyperintensities. Findings in the thalamus were seen in cases 19, 29 and 30 in whom bilateral thalamic hyperintensities, partial fusion of thalami inferiorly and bilateral posterior hyperintensity were seen, respectively.

### Anomalies of the cortex and lobes

Of 11 patients with these anomalies (33.3%), 3 patients had volume loss in their images. Loss of brain parenchyma mainly bi-occipital lobes, volume loss of the right temporal lobe and bilateral frontal lobe volume loss compared to the temporal, parietal and occipital formations observed in cases 22, 23 and 30, respectively. Atrophy was also noted in 2 patients with left fronto-parietal and right frontal atrophy in case 13, and cortical and subcortical atrophy in case 20. Other findings identified include: bilateral frontal lobe hypointensity (case 4), cortical hypodensity with patchy enhancement (case 9), and bilateral cortical band of grey matter with intervening white matter and frontal nodules similar to grey matter (case 10). Case 16 also had bilaterally symmetrical subcortical hyperintensities. Widening of sulci and prominence of gyri over the right cerebral hemisphere (fronto-temporal and parietal), incomplete separation of cerebral hemisphere anteriorly and left middle cerebral artery (MCA) region chronic infarction were observed in cases 23, 29 and 33, respectively.

### Anomalies of the corpus callosum

2 (6.1%) of the patients studied had abnormalities of the corpus callosum. These included thinned corpus callosum in case 12 and partial hypoplasia in case 29.

### Abnormalities of the lateral ventricles

Lateral ventricular pathology was also appreciated in 4 (12.1%) of the patients. Dilated lateral ventricles were observed in cases 9 and 28. Rudimentary falx and right occipital horn of the right ventricle were seen in case 29, whereas a deep right occipital horn posteriorly and temporal/frontal minimal increment in CSF spaces including cisterns were noted in case 31.

### Cysts

6 out of the 33 patients (18.2%) had cysts. 2 of these had non-pathological cysts, which include: arachnoid cyst (case 7) and left choroid fissure cyst (case 19). Pathological cysts were found in 4 of the patients with right temporal proencephalic cyst (case 14) and dorsal interhemispheric cyst (29). Multiple cystic lesions with ring enhancing lesions over the bilateral cerebral hemispheres were seen in case 28 while cystic changes which follow CSF signal intensity sparing only the posterior fossa structures were observed in case 22.

### Abnormalities of the cerebellum

Only one patient (3%) had cerebellar findings with marked paracerebellar atrophy as seen in case 26.

### Miscellaneous/other findings

Other anomalies were observed in 15 (45.5%) of the images including: minimally increased CSF spaces (case 8), dilated 3rd ventricles (cases 9 and 28), polymicrogyria (bilateral frontal lobes and perisylvian fissure) in case 10, and periventricular hyperintensities (case 12). Right parietal old hemorrhage (case 14), dilated prevascular spaces on T2 FLAIR (case 16), and remarkable diffusion weighted imaging (DWI), restrictions/ apparent diffusion coefficient (ADC) hypointensities in the MCA territory (case 17) were also seen. Bilateral periventricular white matter linear T2/T2 FLAIR hyperintensities with no restriction and no post contrast enhancement were found in case 18. Increased subarachnoid space and adjacent T2 hyperintensity were observed in case 20, dilatation of 3^rd^ ventricle with mega cisterna magna in case 22, absent septum pellucidum with squaring of the frontal horns in case 24 as well as 4^th^ ventricle ex-vacuo dilation and dilated CSF cisterns in case 26. Moreover, absent septum pellucidum was seen in case 29 as well and parenchymal volume loss with widening of the interhemispheric fissure and sulci in case 32.

54.5% (18/33) of patients were males with a higher use of MRI 81.8% (27/33). Most of the children in the study had been born at term, 69.7% (23/33). Normal brain scans were seen in 30.3%. (10/33) as opposed to 69.7% abnormal brain scans (23/33) (Table 2).

**Table 2:** Characteristics of pediatric patients with cerebral palsy who underwent neuroimaging at ACSH between January 2016 and August 2019.

**Sex (N = 33)**	**N (%)**
Male	18(54.5)
Female	15(45.5)
Total	33(100.0)
**Imaging modality**	**N (%)**
MRI	27 (81.8)
CT	6 (18.2)
Total	33 (100.0)
**Gestational age at delivery**	
Term	23 (69.7)
Preterm	1 (3.0)
ND	9 (27.3)
Total	33(100)
**Imaging findings**	
Normal	10 (30.3)
Abnormal	23 (69.7)
Total	33 (100)

ND- Not documented; CT- computed Tomography scan; MRI- Magnetic Resonance Imaging

Among the patients studied, normal brain scans were seen equally in patients with hypotonic and hemiplegic/hemiparetic CP, 12.1% (4/33) each. Abnormal scans were observed mostly in patients with spastic quadriplegia, 33.3% (11/33), hemiplegic CP and unspecified forms, 12.1% (4/33), spastic diplegia, 9.1% (3), choreoathetoid CP 6.1% (2/33), and Monoparetic CP, 3% (1), respectively. (Table 3).

**Table 3:** Patterns of neuroimaging findings based on type of CP from January 2016 to August 2019.

CP Type		Finding	
		Normal N (%)	Abnormal N (%)
	Hemiplegic/hemiparetic CP	4 (12.1)	4 (12.1)
	Hypotonic CP	4 (12.1)	0
	Spastic quadriplegia	0	11 (33.3)
	Choreoathetoid CP	0	2 (6.1)
	Unspecified forms	0	4 (12.1)
	Spastic diplegia	0	3 (9.1)
	Monoparetic CP	0	1(3)
	**Total of 33**	**10 (30.3)**	**23 (69.7)**

CP: Cerebral Palsy

Distribution of brain findings among the CP types was varied. Patients with spastic quadriplegia had the most findings in their brain scans with miscellaneous findings and cortex/lobe anomalies being the majority, 24.2% and 15.2%, respectively (Table 4).

**Table 4:** Distribution of Neuroimaging findings based on CP type from January 2016 to August 2019.

Type of CP	Neuroimaging Findings							
	White matter	Basal Ganglia/ Thalamus	Cortex/lobes	Corpus Callosum	Lateral Ventricle	Cyst	Cerebellum	Other findings
	N (%)	N (%)	N (%)	N (%)	N (%)	N (%)	N (%)	N (%)
Hemiplegic/hemiparetic CP	1 (3)	1 (3)	3 (9.1)	0	0	1 (3)	0	2 (6.1)
Hypotonic CP	0	0	0	0	0	0	0	0
Spastic quadriplegia	3 (9.1)	0	5 (15.2)	1 (3)	2 (6.1)	3 (9.1)	1(3)	8 (24.2)
Choreoathetoid CP	0	1 (3)	1 (3)	0	0	0	0	0
Unspecified forms	1 (3)	1 (3)	1 (3)	0	0	1 (3)	0	3 (9.1)
Spastic diplegia	1 (3)	1 (3)	1 (3)	1 (3)	2 (6.1)	1 (3)	0	2 (6.1)
Monoparetic CP	0	0	0	0	0	0	0	0

CP: Cerebral Palsy

## Discussion

In this study, the median age at neuroimaging was 2 years. The minimum age was 3 months and the maximum age was 15 years. This is in line with Sharma and Dhande [1], who found that most of the children were in the age group ranging from 1 to 2 years. In contrast, a higher age of four years was found by Bearden et al. [16] while Moifo et al. [17] reported 42 months with 77.4% aged 0 to 60 months. This depicts early health seeking behaviour among our population. It may also point to the possibility of having had patients with unclear etiology or clinical course that guided clinician inclination to conduct neuroimaging at an earlier age; seeing as majority of the patients had severe diseases.

We also found a higher number of males than females (Table 2) with a male: female ratio of 1.2:1. This is in congruent with most CP studies where male predominance has been demonstrated with, male: female ratios of 1.3–1.4:1 [16, 18, 19]. Chromosomal variants such as recessive X-linked, autosomal and rare autosomal dominant genes have been implicated in this difference. This suggests that males may be more vulnerable to genetic mutation and neurodevelopmental disorders than females [20].

There were also more patients who had been born at term in our study (Table 2), with one patient who had been delivered prematurely at 7 months of gestation. Bax et al. [6] reported that majority (54%) of the population they studied was born at term while 10.9% were very preterm (28 weeks), which is similar to our study. Another support for this finding is a study carried out by Aggarwal et al. [21] in which only 22.2% of 98 children diagnosed with cerebral palsy at a tertiary center were preterm. In contrast, a Norwegian study identified CP having a higher association with preterm deliveries [22]. The high number of patients born at term in our study is concordant with a Ugandan study where there were more term patients and those born preterm were also quite low. This was associated with poor preterm infant survival rates [9]. This may allude to a lower rate of survival for preterm patients with complications that would result in CP even in our setup as a similar low income country. A number of the patients’ files reviewed had no documentation on gestational age at delivery. This may be attributed to patients who came with caregivers other than their mothers and may not have this information; patients born in peripheral hospitals who were referred to ACSH without their accompanying documented birth history, and possible health workers’ documentation error.

**Fig 1:**
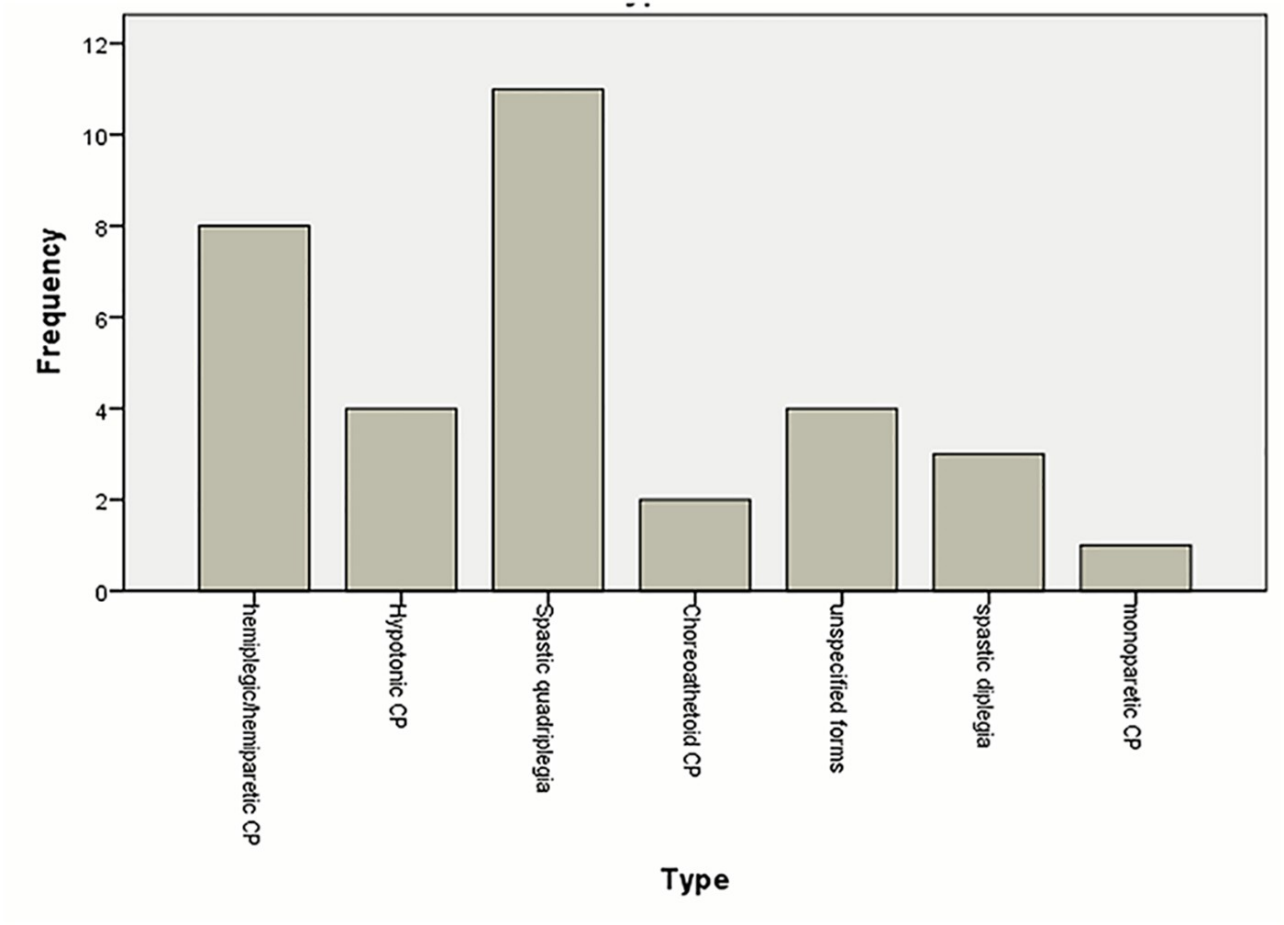
Types of cerebral palsy of patients who underwent neuroimaging at ACSH between January 2016 and August 2019. Majority of the patients had spastic quadriplegia (33.3%) followed by spastic hemiplegia/hemiparesis (24.2%) and hypotonic CP (12.1%). 9.1% of the patients had spastic diplegia while patients with choreoathetoid and monoparetic CP constituted 6.1% and 3% each. 12.1% of the patients had unspecified forms.

We found that spastic forms of CP were more common than dyskinetic forms with the majority being spastic quadriplegia (Fig 1). Patients with spastic quadriplegia also had the highest number of findings in their brain scans compared to other types (Table 4). Similar findings were reported in Botswana where the authors expressed that spastic CP was the most preponderant of the CP types; the most prevalent being spastic quadriplegia followed by spastic hemiplegia and diplegia [16]. Another study conducted in Jos, Nigeria, identified spastic CP as having the highest occurrence among patients studied [23]. A Swedish study had divergent view, where hemiplegia (44%) was the most common type, followed by diplegia then quadriplegia [2]. Spastic quadriplegic CP is often caused by insults that cause neuronal loss to the cortex [3]. Hypoxic ischemic encephalopathy is one of the main predisposing risk factors that results in both spastic quadriplegia with cortical lesions [6]. We, therefore, postulate that the high number of patients with spastic quadriplegia in our study may reflect a higher incidence of hypoxic ischemic encephalopathy in our setup. Further research to elucidate this hypothesis is recommended.

The use of MRI was also higher than CT scan for CP patients in our study. This conforms to an American study conducted by Wu et al. [24] where more CP patients studied had undergone MRI as opposed to CT scans. MRI is preferred to CT scan in the neuropathology of CP as MRI is sensitive in detecting PVL, other perinatally acquired lesions and subtle congenital anomalies of brain development with an accuracy of as high as 70–90% in various studies [25, 26]. It is the imaging modality of choice among older children (as early as above 2–3 weeks of life). This is because it defines cortical and white matter structures and abnormalities more clearly than any other method. It also allows for the determination of appropriate myelination for a given age and may have a role in predicting neurodevelopmental outcomes in preterm infants [27, 28]. However, neuroimaging findings still pose a challenge in children of age less than 2 years where there is still a high possibility of missing pertinent findings [29]. In some studies where MRI and CT scan imaging have been compared in CP patients, it was noted that CT scans were less likely to identify various anomalies such as white matter pathology [25]. We advocate for more scanning and reduction in MRI costs in patients who have a diagnosis of CP, especially with unclear clinical course or etiology. This would facilitate the identification of causative factors and differentiation from other neurological conditions with similar manifestations in the population. This may also aid in the establishment of appropriate preventive strategies in the presence of evidence based etiology.

Almost one-third of the images studied were normal in patients having hypotonic, spastic hemiplegic and spastic quadriplegic CP as seen in Table 3. All but one of the patients with normal findings had been investigated with MRI. Normal neuroimaging findings in patients who fulfill the clinical criteria for CP have also been highlighted in several other studies [20, 26]. These associated normal MRIs with lack of perinatal adversity as well as with the dyskinetic, ataxic/ hypotonic and spastic diplegic CP subtypes. This is similar to our findings where more than half of the normal images were of patients with hypotonic and dyskinetic CP. The high number of normal scans in our study may be due to younger age at imaging (patients less than two years of age). Brain structure continues to change rapidly during early childhood. Any abnormality may, therefore, not be apparent until 2 years of age as maturation of the myelination process and development of the deep grey structures may be less obvious until around this time [29].

**Fig 2:**
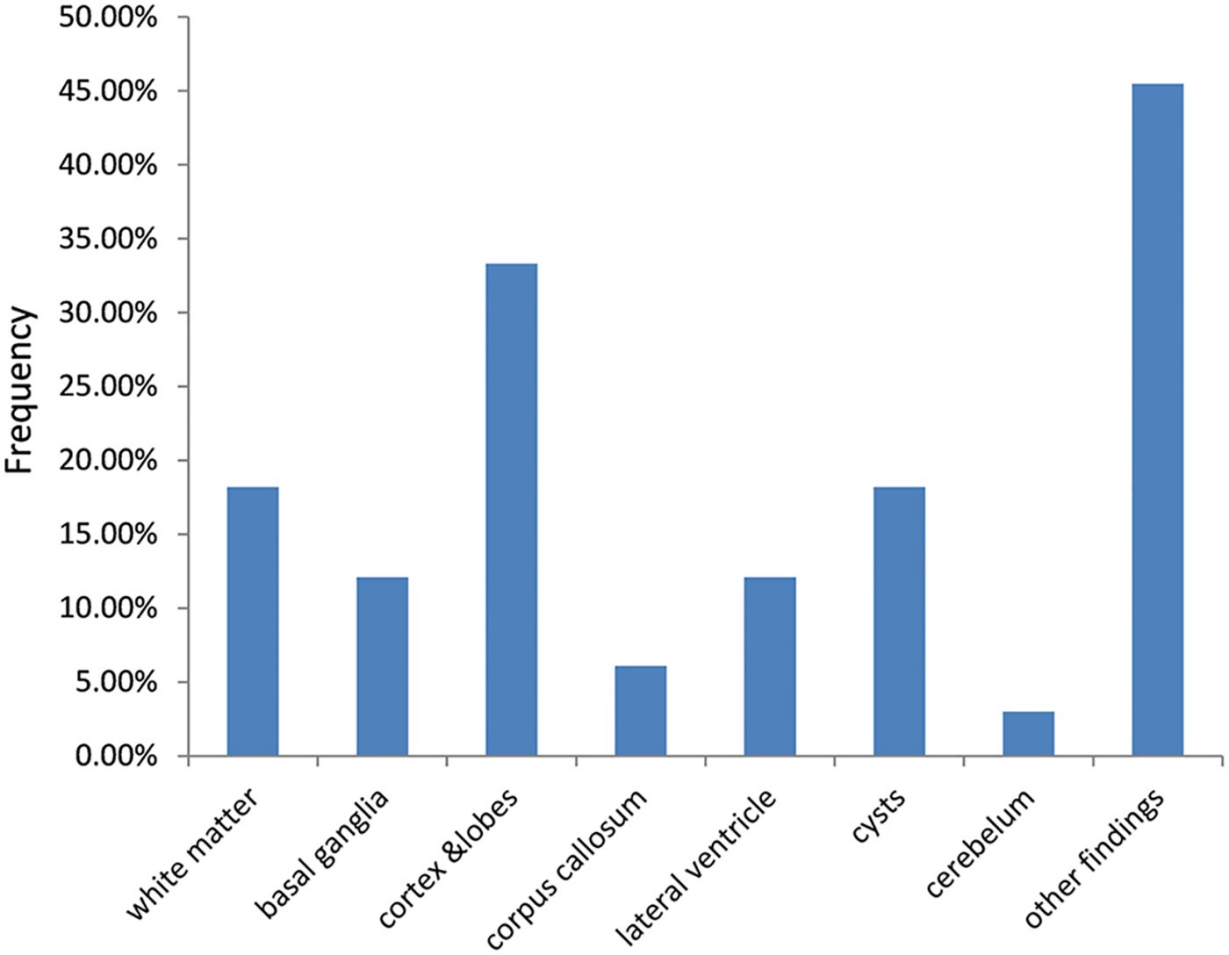
Distribution of neuroimaging pathology among pediatric patients with cerebral palsy from January 2016 to August 2019. Neuroimaging anomalies included pathologies of the white matter (18.2%), basal ganglia (15.2%), cortex and lobes (27.3%), corpus callosum (6.1%), lateral ventricle (12.1%), cysts (18.2%) and cerebellum (3%), respectively. Other findings were found in 45.5% of the patients.

**Fig 3:**
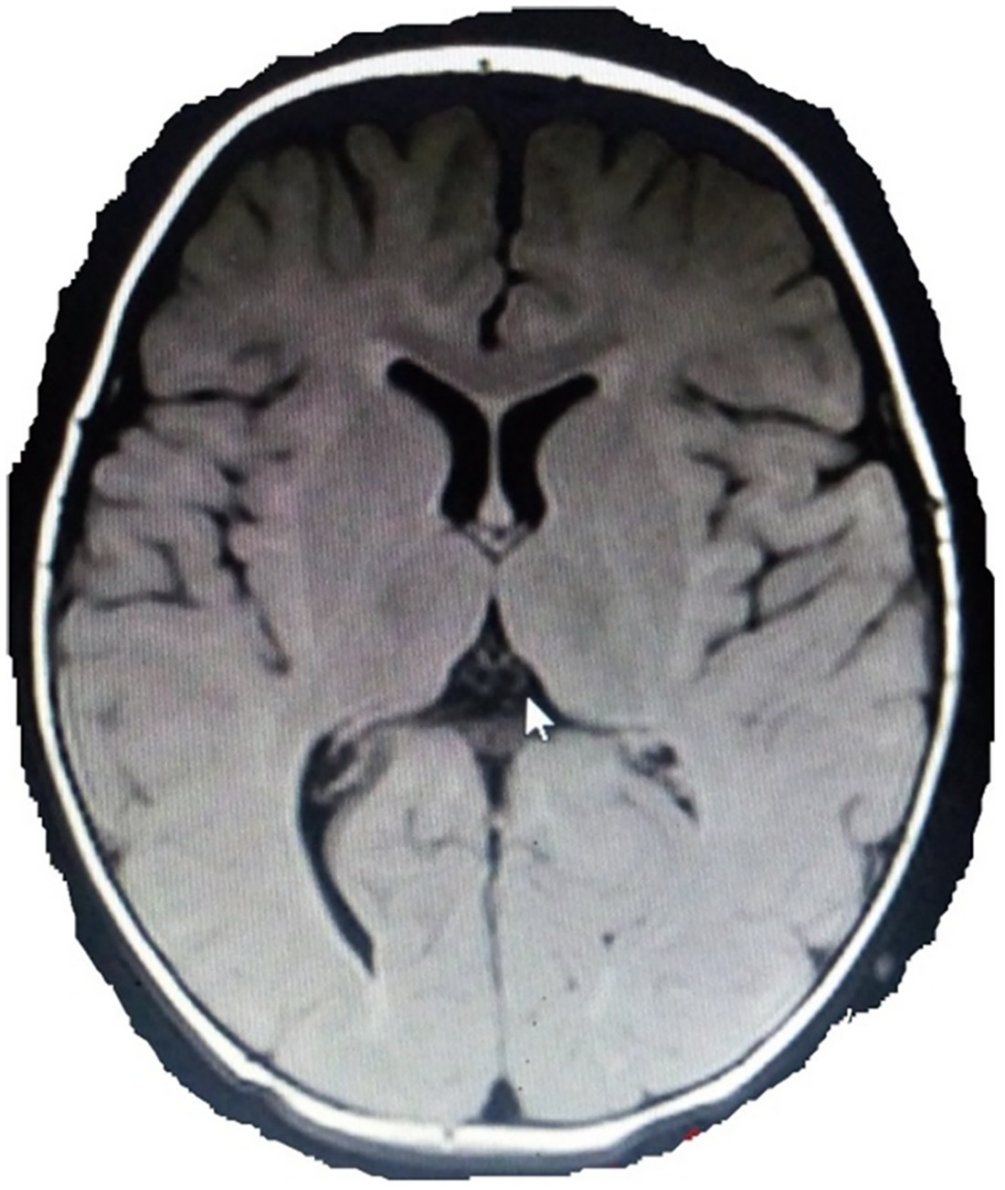
MRI of an 11 month old patient with spastic diplegia. MRI of an 11 month old male patient born at term with spastic diplegia (Case 31**)** showing deep right occipital horn posteriorly with temporal/frontal minimal increment in CSF spaces including cisterns (T2 FLAIR).

**Fig 4:**
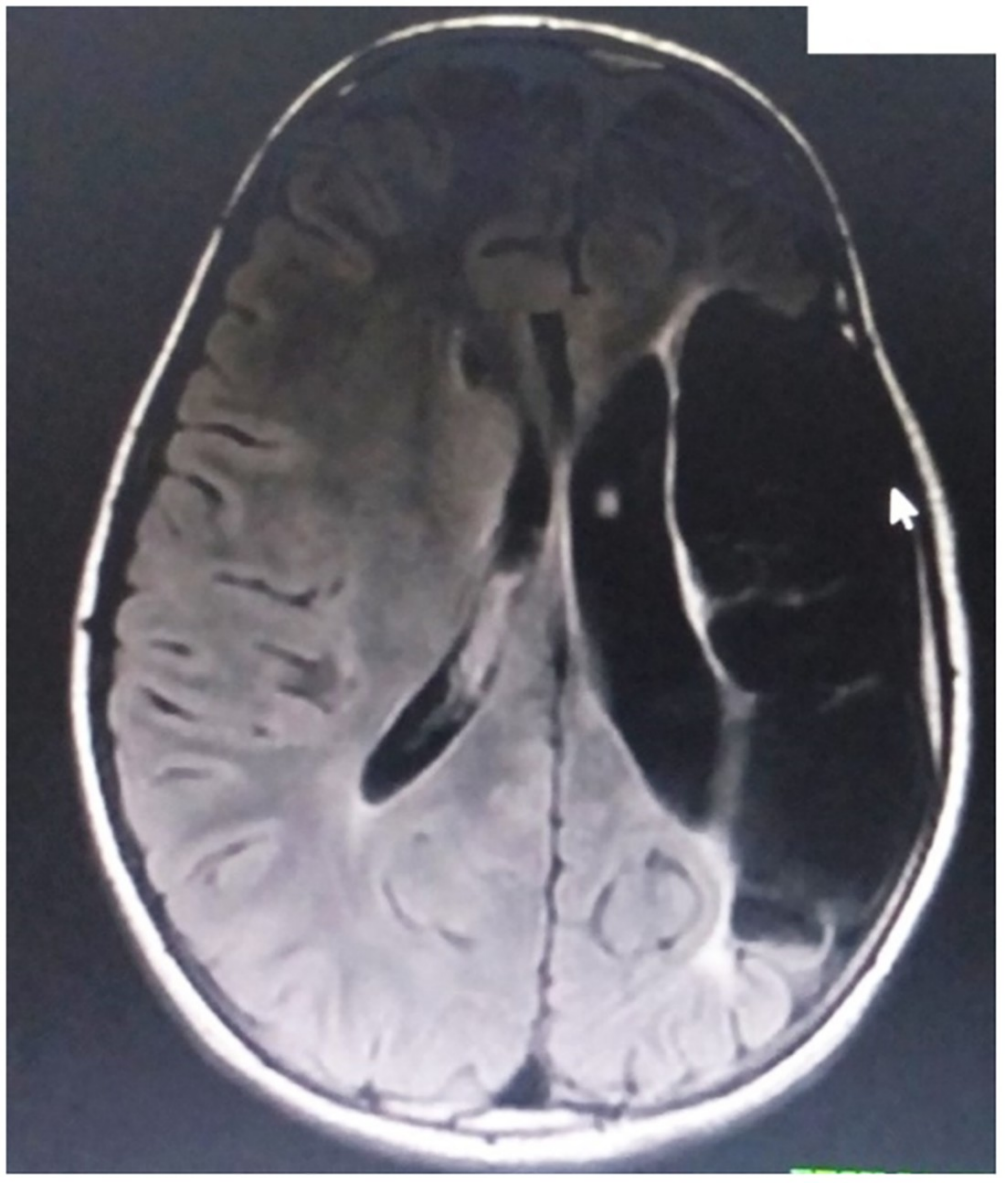
MRI of a 13 year old with right spastic hemiparesis. MRI of a 13 year old male patient born at term with right spastic hemiparesis (Case 20) showing cortical and subcortical atrophy with increased subarachnoid space and adjacent T2 hyperintensity; most likely due to a perinatal stroke.

**Fig 5:**
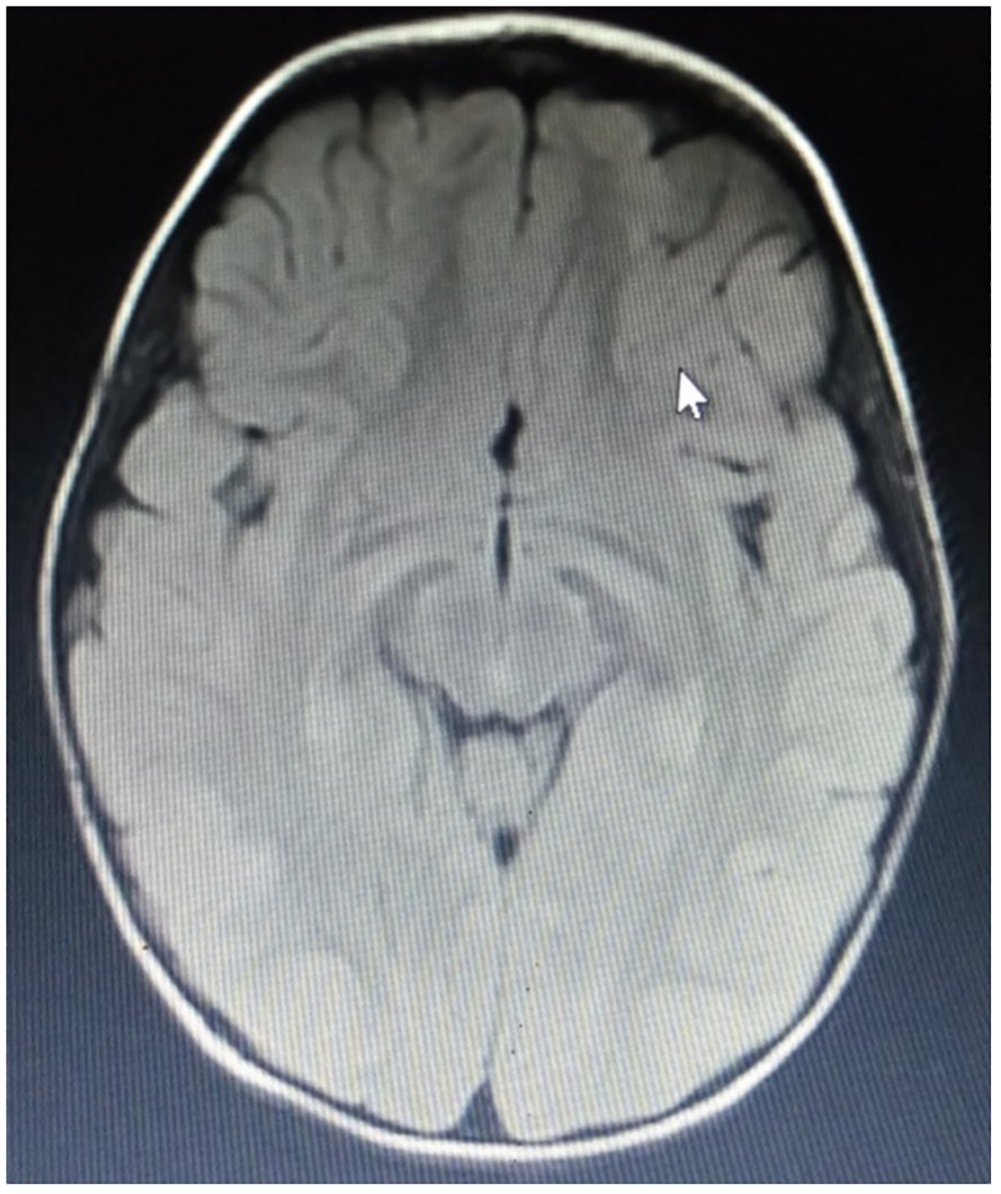
Case 30: MRI of a 3 year old patient with choreoathetoid CP. MRI of a 3 year old female patient born at term presenting with choreoathetoid CP, showing bilateral frontal lobe volume loss compared with the temporal-parietal and occipital formations and Bilateral basal ganglia posterior hyperintensity on axial T2 FLAIR.

**Fig 6:**
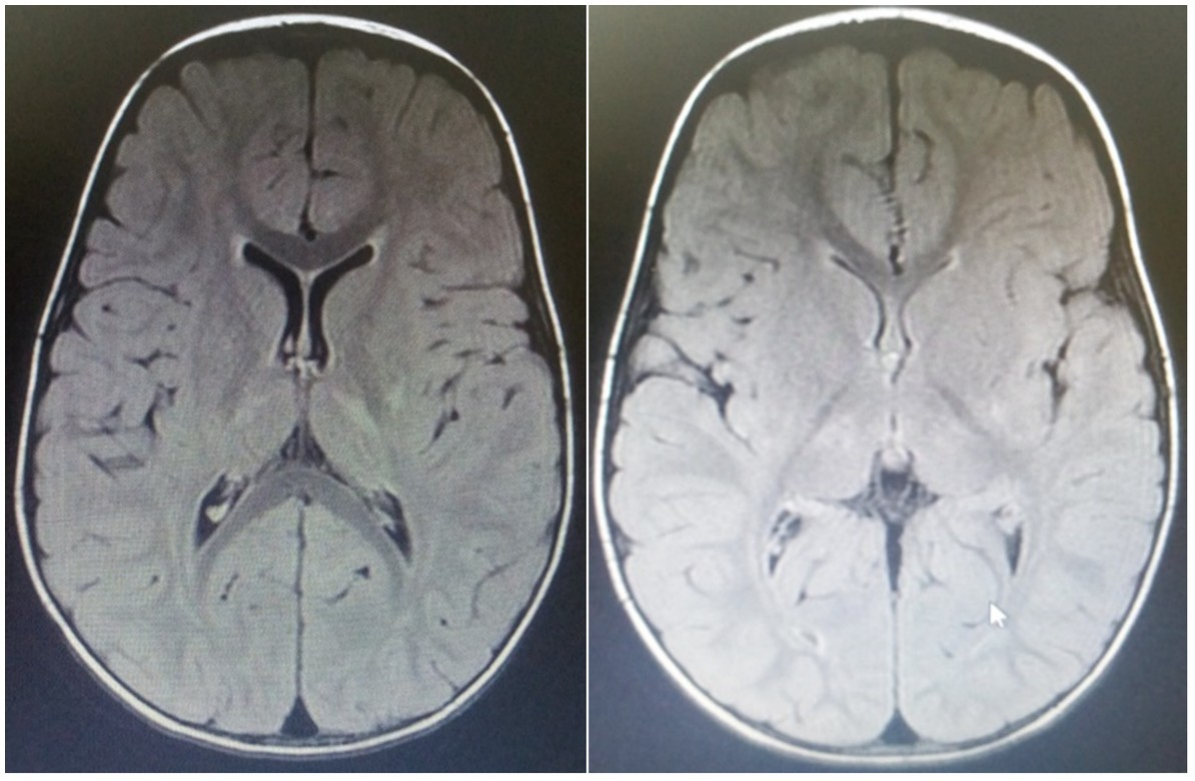
Case 19: MRI of a 3 year old male patient with unspecified form. MRI images of a 3 year old male child delivered at term who did not cry immediately after birth. Axial T2 FLAIR MR images showing bilateral basal ganglia and thalamic hyperintensities.

Pathologies were classified into white matter anomalies, basal ganglia and thalamus, cortex and lobes, lateral ventricles, cysts, corpus callosum and cerebellar anomalies as well as other findings as seen in Fig 2. Examples of these findings are seen in Figs 3–6. The most common abnormalities were found in the cortex and lobes observed mostly in patients with spastic quadriplegia. These patients also had the highest number of other associated findings. This is in agreement with Yin et al. [30] who found more cortical malformations in their work than white matter lesions. A different finding, is presented by Towsley et al. [5] who observed in their research that majority of their patients had periventricular white matter injury. Bax et al. [6] also demonstrated white matter injury as the most common imaging pattern, followed by grey matter injury. Due to selective vulnerability, causative factors between 24 and 34 weeks of gestation involve immature oliogdendroglia which are especially susceptible to injury, while in hypoxic ischemic encephalopathy (HIE) in full-term infants and kernicterus, the primary vulnerability is in deep gray structures [25]. Our findings, therefore, may be due to the higher number of patients born at term, as the white matter lesions are more frequent in preterm infants who were quite few in this study.

## Conclusion

Our study revealed cortical and subcortical brain injury as the most common anatomical feature seen in the MRI and CT scan images of pediatric CP patients in ACSH, Mekelle. Spastic quadriplegia was the most occurring form of CP. Brain imaging may help in identifying timing of brain insults and possible CP etiologies in low-income countries like Ethiopia. Therefore, enabling universal brain scanning for CP patients may serve in aiding future creation and implementation of possible preventive interventions for this lifelong disorder.

## Supporting information

S1 Dataset (SAV)
